# The mediating role of trait emotional intelligence in the relationship between parental neglect and cognitive emotion regulation strategies

**DOI:** 10.1186/s40359-024-01817-3

**Published:** 2024-05-30

**Authors:** Utku Beyazit, Yeşim Yurdakul, Aynur Bütün Ayhan

**Affiliations:** 1 Kumluca Health Sciences Faculty, Child Development Department, Akdeniz University, Temel Eğitim Mah. Spor Cad. No: 14, Kumluca, Antalya Turkey; 2 Faculty of Health Sciences, Child Development Department, Ankara University, Tepebaşı, Fatih Cd. No:197/A, Keçiören, Ankara PK: 06290 Turkey

**Keywords:** Trait emotional intelligence, Neglect, Cognitive emotion regulation strategies

## Abstract

**Background:**

Examining children’s abilities to recognize and regulate their emotions in the context of parental neglect is of significant importance in order to comprehend the dynamics of and to support the development of emotional skills of children, particularly those at risk of neglect. From this point of view, the aim of the study was to examine the mediating role of trait emotional intelligence (trait EI) in the relationship between parental neglect and cognitive emotion regulation strategies (CERS) in children.

**Method:**

The study group consisted of 265 children (135 girls and 130 boys) who were attending two separate primary schools in the city center of Antalya, Turkey. The mean age of the children was 10.27 ± 0.45. As the data gathering instruments, an “Individual Information Form” was administered to assess the socio-demographic information of the children, while the “Multidimensional Neglectful Behavior Scale-Child Report was administered to examine the level of neglect of children by the parents, the “Trait Emotional Intelligence Questionnaire-Child Form” was administered to assess the trait emotional intelligence level, and the “Cognitive Emotion Regulation Strategies for Children Scale” was administered to assess the CERS of the children.

**Result:**

It was found that trait EI played a full mediator role in the relationship between CERS and both maternal and paternal neglect (*p < .05*), except for the relationship between paternal neglect and maladaptive CERS (*p > .05*).

**Conclusions:**

The results may suggest that neglected children use all emotion regulation skills, including both adaptive and maladaptive, to cope with their negative emotional experience, but may use adaptive CERS more if their trait EI is higher.

## Introduction

Emotional intelligence is defined as the ability to perceive, display, interpret, control, evaluate and use emotions to communicate and relate effectively and constructively with others [1, 2]. Goleman [3] suggested that emotional intelligence consists of components of social and emotional domains. According to this approach, while empathy and social skills in interpersonal relations are included in the domain of social competence, the concept of emotional regulation is included in the emotional domain. According to Petrides and Furnham [4], there are two different types of emotional intelligence, namely ability and trait EI. According to this classification, while ability EI refers to the actual ability of individuals to process and use emotion-related information [5], trait EI refers to the self-perception and behavioral aptitude regarding individual processing and using emotional information [6]. According to this definition, trait EI is a construct consisting of self-perceptions and dispositions related to utilizing emotion-related information [7]. In this respect, it can be thought that trait EI may be more related to the individual’s psychological traits and cognitive strategies. However, it can also be suggested that cognitive and emotional dispositions and skills related to social competence should operate together in order for individuals to understand and express themselves effectively, to understand and relate to others, and to cope with daily demands successfully.

Emotional experiences are formed by a person’s self-perception and cognitive interpretation of their experiences. In other words, individuals largely determine their emotions through cognitive interpretation or evaluation of their experiences [8]. The individual’s cognitive evaluations determine the emotions and the way they will be experienced. In this context, the concept of cognition emerges as the main way in which emotions are regulated [9]. Before individuals exhibit an emotional reaction to a stressful event, they first process the situation they encounter through cognitive processes and then adjust their emotional response accordingly [10]. Consequently, as regulation skills emerge through the interaction of various processes such as cognition, social skills, empathy, and recognizing understanding and managing emotions, a high level of cognitive control is required for the successful regulation of emotions [11, 12].

On the other hand, difficulties that arise in the components of ability emotional intelligence related to personal and social competences may cause difficulties for individuals to adapt to social conditions, use their communication skills, achieve success in relationships by establishing effective communication, and act by taking into account the emotional state of themselves and the other person. This, in turn, may lead to deficiencies and problems in the cognitive and psycho-social skills of regulating emotions [13, 14]. In various studies, it has been suggested that children with higher trait EI who can make sense of and regulate their emotions display less emotional psychopathologies [15–18]. In a number of other studies, significant relationships were found between trait EI and a more stable self-esteem [19], socio-emotional competence [20] and behavioral problems [21] in children. The common aspect these findings is that they reveal that emotion regulation has extremely complex effects, and it is of great importance in terms of the ability to cope with negative situations and maintain positive situations, which are necessary for psycho-social functionality. This argument also overlaps with the approach of Garnefski et al. [22], who conceptualized emotion regulation in the same category as cognitive coping and introduced the concept of CERS. Garnefski et al. [23] argued that some of these strategies are adaptive and contribute to the social and psychological functionality of the individual, while others are maladaptive and dysfunctional. Adaptive strategies include accepting the event experienced, focusing on more positive issues instead of thinking about the negative aspects of the event, thinking about how to handle the negative situation and what to do next, while maladaptive strategies include blaming oneself or others for a negative experience, catastrophizing and ruminative thinking about the negative events [24, 25]. While adaptive strategies make it easier to solve interpersonal problems, maladaptive strategies weaken individuals’ sense of control and make it difficult for effective coping skills to emerge [26, 27].

The strategies that will be used and the functionality of these strategies are shaped by developmental experiences and show individual differences [28]. In order to understand the dynamics of these strategies, it is crucial to examine their developmental patterns and the reasons why some strategies are more effective than others [29]. Emotion regulation strategies that emerge at an early age are generally not based on cognitive processes, and children can only master the ability to use cognitive strategies to regulate emotions and to choose appropriate ways of regulating them as they become older [30, 31]. Thus, in the transition from early childhood to middle childhood, children’s capacity to regulate their own emotions increases qualitatively. Along with age and developmental maturity, family environment is one of the most important factors affecting the emotional and cognitive development process of the child. The family teaches the child the norms that determine how and when to express emotions, what to feel and how to behave in social situations [32]. When family-related factors involve developmental risks, they can lead to difficulties in the development of emotional competencies as well as maladaptive behavior patterns [33, 34].

At the forefront of these risk factors is child maltreatment, particularly the concept of neglect, which is defined as the failure or inadequacy of the care givers in meeting the child’s basic needs such as nutrition, security, education, shelter and health [35]. Neglectful parents communicate less about emotions in their relations with their children, and also have problems in perceiving and interpreting emotional messages and emotional cues from the child, approaching the child with empathy, responding to the child appropriately, and creating an adequate environment for emotional socialization [36]. As a result, neglect may lead to a wide variety of negative consequences ranging from developmental delays to mental and behavioral problems [37]. Various studies have revealed that neglected children have attachment [38, 39] and behavioral problems [40, 41], exhibit difficulties in cognitive and social skills [42, 43], have low levels of empathy [44, 45] and emotional self-awareness [46], have poor understanding of emotions such as anger and sadness [47], and experience social adjustment difficulties [48, 49].

Although it has been revealed that neglected children experience cognitive, emotional and social problems, no studies have focused on the associations between parental neglect, children’s trait EI levels and CERS. Various studies have shown that the maltreatment of children in general affects emotional intelligence negatively. However, these studies were conducted on adolescent [50, 51] or mostly on adult samples [52–54]. Furthermore, these studies were mostly related to the concept of emotional intelligence whereas it is suggested that as a personality disposition trait EI is more related to parental behaviors [55]. In this respect, the relationship between neglect and trait EI remains a subject that requires attention. On the other hand, an examination of relationship between parental neglect and emotion regulation revealed that the exposure to parental neglect in early childhood causes difficulties in the development of emotion regulation [56]. In a meta-analysis including 35 studies, it was also determined that the maltreatment of children in general had negative effects on the development of emotional coping skills [57]. The fastest period of development in terms of emotional intelligence is between the ages of five and ten [58]. While children dominantly use behavioral strategies until the age of five when regulating their emotions, the development of the cognitive dimension of emotion regulation accelerates from the age of five to adolescence [59]. From this point of view, it can be argued that primary school years are important in terms of cognitive processes related to trait EI and emotional regulation. The effect of neglectful attitudes on these processes also begins to become evident as difficulties emerge in the emotional skills of children during the school years [60]. In this respect, examining children’s abilities to recognize and regulate their emotions in the context of parental neglect is of significant importance in order to comprehend the dynamics of and to support the development of emotional skills of children, particularly those at risk of neglect. From this point of view, the aim of this study was to examine the mediating role of trait EI in the relationship between parental neglect and CERS in children. According to the theoretical assumptions and the literature findings discussed above, since parental neglect impedes higher trait EI, it was hypothesised that both maternal and paternal neglect would negatively predict trait EI. Furthermore, it was hypothesised that neglect would predict both adaptive and maladaptive CERS and that these associations would be mediated by trait EI.

## Method

### Participants

The study group of the research consisted of 265 children who were attending two separate primary schools in the city center of Antalya, Turkey. While forming the study group, the list of elementary schools in the center of Antalya was provided from the Provincial Directorate of the Ministry of National Education. In addition, the data on the socioeconomic level of the districts where the schools are located were provided by the Turkish Statistical Institute (TUIK). Based on TUIK data and the opinions of the Provincial Directorate of National Education, the two schools the study was planned to be implemented were selected from those with a heterogeneous population structure and socio-economically represented the city in general. The mean age of the children included in the study was 10.27 ± 0.45. With regard to their gender, 135 (50.9%) of them were girls and 130 (49.1%) of them were boys. Regarding the income level of the families of the children, 9 (3.4%) had low income, 167 (63%) had average income, and 89 (33.6%) had high income.

### Instruments

In the study, an “Individual Information Form” was administered to assess the socio-demographic information of the children while the “Multidimensional Neglectful Behavior Scale-Child Report” scale was administered to examine the level of neglect of the children by their parents, the “Trait Emotional Intelligence Questionnaire-Child Form” was administered to assess the trait emotional intelligence level and the “Cognitive Emotion Regulation Strategies for Children Scale” was administered to assess the CERS of the children.

#### Individual information form

The form was prepared by the researchers in order to acquire information about the children’s age, gender, grade, and their parents’ income level.

#### Multidimensional neglectful behavior scale-child report

The scale was developed by Kantor et al. [61] to assess the level of neglect of children aged 10–15 by their parents. The scale has two forms, the Mother and Father Form, in which children evaluate the neglectful behaviors of their mothers and fathers separately. Both the original Mother and Father Forms of the scale consist of a total of 66 pictorial cards. Each of the picture cards includes drawings to evaluate children’s neglect experiences by their parents. In the administration, children are shown each pictorial card in an order. On each of the cards, there are two drawings depicted side by side regarding a domain of neglect. One of the drawings depicts a child who is neglected and the drawing on the other side of the card portrays a child whose mother/father is nurturing. The children are shown the card and asked which child in the card they resemble. The responses of the children that identify parental neglect are assigned one point. The Cronbach’s alpha coefficients of the original form of the scale was determined to be 0.78. The Turkish adaptation of the instrument was made by Beyazıt and Bütün Ayhan [62]. In the study, the coefficients for the internal consistency were determined to be 0.83 for the Mother Form and 0.91 for the Father Form. In the present study, the Cronbach’s alpha was computed as 0.78 for the Mother Form and 0.89 for the Father Form.

#### Trait emotional intelligence Questionnaire–Child form

The scale was developed by Mavrovelli et al. [63] to assess the trait emotional intelligence of children aged 8–12. It consists of two distinctive factors and 75 five-point Likert type items with responses ranging from (1) strongly disagree to (5) strongly agree. The scale assess the socio-emotionality and emotional control of children in general. The concept of socio-emotionality refers to children’s emotional experiences as well as their ability to express their emotions and understand the emotions of other people, while emotional control refers to children’s ability to regulate and manage their own behaviors and emotions. In the reliability study of the original scale, the coefficient for internal consistency was found to be 0.76 [68]. The scale was adapted to Turkish by Beyazıt et al. [64] and the Cronbach’s alpha coefficient of the Turkish version was found to be 0.91. In the present study, the coefficient for internal consistency was computed as 0.89.

#### Cognitive emotion regulation strategies for children scale

The scale was developed by Garnefski et al. [65] to assess the CERS of children aged 9–11. It consists of 36 five-point Likert type items with responses ranging from (1) never to (5) always. The scale assesses the strategies of rumination, acceptance, positive refocusing, refocusing on the plan, positive reappraisal, putting into perspective, catastrophizing, self-blame, and blaming others. These strategies are divided into two basic dimensions, namely adaptive and maladaptive CERS. Acceptance, positive refocusing, positive reappraisal, refocusing on the plan, and putting into perspective are classified as adaptive coping strategies, while rumination, catastrophizing, blaming oneself and others are classified as maladaptive coping strategies. Scores from each dimension indicate the level of children’s coping strategies in that dimension. In the reliability study of the scale conducted by Garnefski et al. [65], Cronbach’s alpha coefficients were found to vary between 0.67 and 0.97. The scale was adapted to Turkish by Akfrat and Turan [66] and the Cronbach’s alpha coefficient of the Turkish version of the scale was determined to be 0.79. In the present study, the Cronbach’s alpha coefficients were found to be 0.78 for the adaptive coping strategies dimension and 0.71 for the maladaptive coping strategies dimension.

### Procedure

In the study, permissions to administer the data gathering instruments from the authors of the scales and ethical board approval of the Humanitarian Research Ethics Committee (*track code is blinded for review*) were initially obtained. Subsequently, school administrators and classroom teachers were given information about the study, and necessary permissions were obtained to conduct the research in the schools. In the process of administration, children were given informed consent forms to be forwarded to their families. In the forms, explanations were made about the content of the study and the parents’ consent was sought for their children’s participation in the study. One week later, the schools were revisited and the forms brought to school by the children were collected. Prior to the administration of the forms, the children were also informed about the study and their assent was obtained for their participation. Informed consent was also obtained from the parents of all children included in the study. The Multidimensional Neglect Behavior Scale was first administered to the children by a researcher in small groups, during the study hours, in an empty classroom provided by the school administration. The responses of the children were recorded on scoring sheets. After the pictorial card test was completed, a short break was taken and self-report forms were administered to the children. The forms completed by each child and the response sheets of the pictorial test were matched by giving anonymous codes. The administrations lasted between 30 and 45 min. In conclusion, the forms were implemented to a total of 269 children. All procedures performed in studies involving children were conducted in accordance with both the ethical standards of the research ethics committee and the 1964 Declaration of Helsinki and its subsequent amendments or comparable ethical standards. Prior to the onset of the analysis, the forms of four children were excluded from the study due to the large number of omitted items, and as a result, 265 forms in total were ultimately included in the study.

### Data analysis

In the study, process analyses were conducted to examine the mediating role of trait EI in the relationship between parental neglect and CERS. In this context, linear regression analysis was performed to examine the inter-scale effectiveness. Initially, preliminary analysis was conducted and the residual statistics were investigated to examine whether the data showed a parametric distribution. As a result, the examination of the regression standardized residuals showed that the distribution was close to normal. Since the neglect scores were assessed separately for mothers and fathers, separate models were created in the analysis as maternal and paternal. In the analysis, neglect scores were included in the model as predictor (independent) variables. CERS was included as the outcome (dependent) and trait EI was included as the mediator variable. In the regression analysis, neglect and trait EI were included as the predictor variables in the first and second blocks, respectively. Subsequently, path diagrams for the tested models were created. In the mediation analyses, the significance of the indirect effect was tested by structural equation modeling using the Bootstrapping method (with 5000 samples at 95% confidence level). Prior to testing the models, the children’s age and gender were examined to determine whether they predicted the neglect, trait EI and CERS by using regression analysis. It was seen that none of these potential confounding variables significantly predicted the study variables, and hence, they were not taken into consideration in subsequent analyses. SPSS 23 and AMOS 21 programs were used for the statistical analyses.

## Results

The descriptive statistics and correlations between the measures administered in the study are shown in Table 1 below.

**Table 1:** Descriptive statistics and pearson rank correlation coefficients results

	*M*	*SD*	1	2	3	4
1. Maternal neglect	2.29	2.62	-			
2. Paternal neglect	4.03	4.56	0.588**	-		
3. Trait EI	264.40	31.53	− 0.337**	− 0.269**	-	
4. Adaptive CERS	64.36	11.60	− 0.128*	− 0.124*	0.420**	-
5. Maladaptive CERS	43.35	8.84	0.126*	0.045	− 0.162**	0.397**

*Note* *=*p* < .05; **p < .01

As shown in Table 1, maternal and paternal neglect scores were significantly and positively correlated with each other (*r = .588, p < .01*) and trait EI was negatively correlated with both maternal (*r*=-.337, *p* < .01) and paternal neglect (*r*=-.269, *p* < .01). Furthermore, adaptive CERS was negatively correlated with maternal (*r=*-.128, *p* < .05) and paternal neglect (*r=*-.124, *p* < .05) and positively correlated with trait EI (*r =* .420, *p* < .01), while maladaptive CERS was positively correlated with maternal neglect (*r =* .126, *p* < .05) and negatively correlated with trait EI (*r=*-.162, *p* < .01). Finally, adaptive and maladaptive CERS were positively correlated (*r =* .397, *p* < .01).

The path estimates related to the mediating role of trait EI are presented in Table 2.

**Table 2:** Path estimates in the model of neglect and CERS mediated by Trait EI

Paths	Std. β	t	*p*	β (95% CI)
Maternal model				
Direct relationship				
Neglect→ Trait EI	-0.337	-5.799	0.001**	-4.051 (-5.427, -2.676)
Neglect→ Adaptive CERS	-0.128	-2.087	0.038*	-0.565 (-1.098, -0.032 )
Neglect→ Maladaptive CERS	0.126	2.066	0.040*	0.426 (0.020, 0.833)
Mediating effect via trait EI				
Neglect→ Adaptive CERS	0.016	0.264	0.792	0.070 (-0.449, 0.588)
Trait EI→ Adaptive CERS	0.420	7.515	0.001**	0.155 (0.114, 0.195)
Neglect→ Maladaptive CERS	0.081	1.254	0.211	0.273 (-0.156, 0.702)
Trait EI→ Maladaptive CERS	-0.162	-2.666	0.008**	-0.045 (-0.079, -0.012)
Paternal model				
Direct relationship				
Neglect→ Trait EI	-0.269	-4.535	0.001**	-0.039 (-0.056, -0.022)
Neglect→ Adaptive CERS	-0.124	-2.024	0.044*	-0.315 (-0.621, 0.009)
Neglect→ Maladaptive CERS	0.045	0.723	0.470	0.086 (-0.149, 0.321)
Mediating effect via trait EI				
Neglect→ Adaptive CERS	-0.011	-0.197	0.792	-0.029 (-0.321, 0.362)
Trait EI→ Adaptive CERS	0.420	7.515	0.001**	1.143 (0.844, 1.443)
Neglect→ Maladaptive CERS	0.001	0.014	0.988	0.002 (-0.240, -0.243)
Trait EI→ Maladaptive CERS	-0.162	-2.666	0.001**	-0.045 (-0.079, -0.012)

*Note* *=*p* < .05; **p < .01

**Fig. 1:**
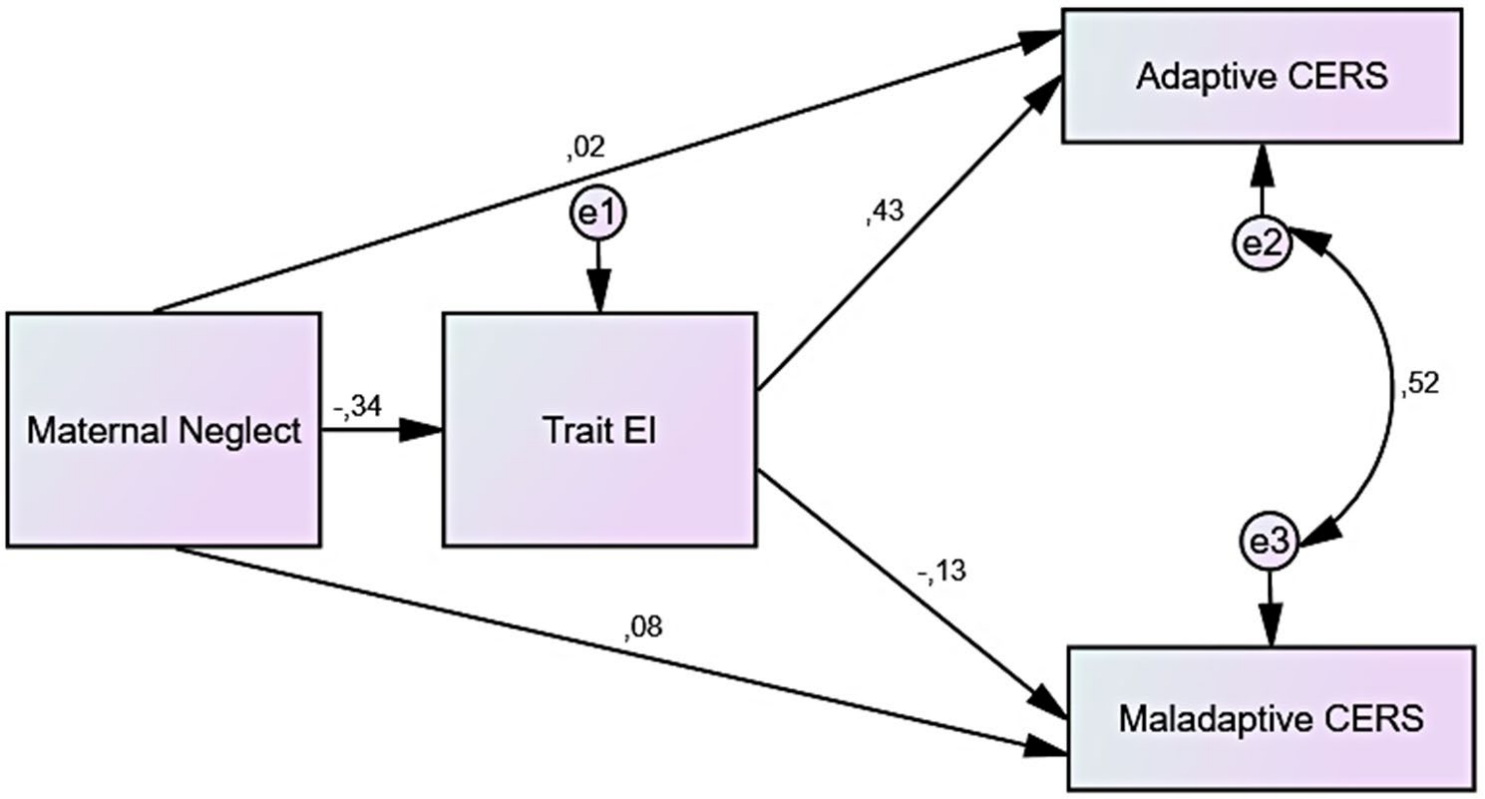
Path diagram of the maternal model

As seen in Table 2, the maternal and paternal models were tested separately in the analysis. When the direct relationships between the variables were examined, it was found that maternal neglect was negatively related with trait EI (β=-0.337, *p* = .001), while in both models, trait EI was positively related with adaptive CERS (β = 0.420, *p* = .001) and negatively related with maladaptive CERS (β=-0.162, *p* = .008). When the relationship between neglect and adaptive CERS was examined, it was found that the direct relationship between the two variables was negative and significant (β=-0.128, *p* = .038), while it became insignificant when the pattern was tested via trait EI (β = 0.016, *p* = .792). While the effect of trait EI is significant, the fact that maternal neglect becomes insignificant shows that trait EI has a full mediator effect on the relationship between maternal neglect and adaptive CERS (F_2,262_= 28.173, *p* < .01, *R* = .421, R^2^ = 0.177). On the other hand, as in the adaptive CERS, while the direct relationship between neglect and maladaptive CERS was positive and significant (β = 0.126, *p* = .040), it became insignificant when the pattern was tested via trait EI (β = 0.081, *p* > .211). The impact of trait EI is significant, however, the fact that maternal neglect becomes insignificant demonstrates that trait EI has a full mediator effect on the relationship between maternal neglect and maladaptive CERS, as evidenced by the analysis with adaptive CERS (F_2,262_= 4.348, *p* < .05, *R* = .179, R^2^ = 0.025). The fit indices for the final mediation model of maternal neglect (א^2^/df = 29.165, *p* < .01; GFI = 0.997, AGFI = 0.983, NFI = 0.990, RFI = 969, CFI = 1, RMSEA = 0.000) showed a good fit of the data to the model [67, 68]. The path diagram created for the full maternal model is presented in Fig. 1.

**Fig. 2:**
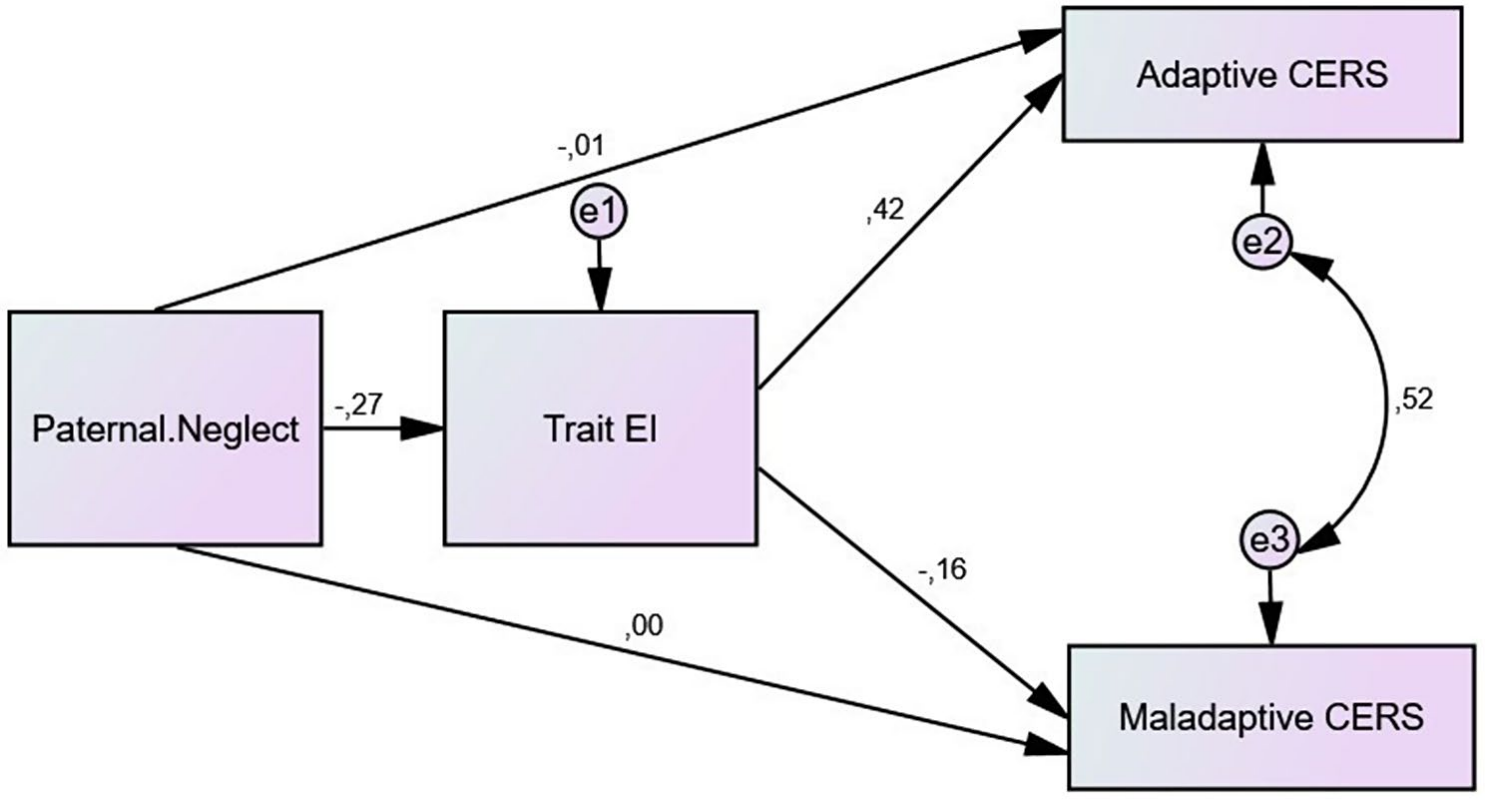
Path diagram for the paternal model

When the paternal model is examined, it is seen that paternal neglect was negatively correlated with trait EI (β=-0.269, *p* = .001) and adaptive CERS (β=-0.124, *p* = .044). However, unlike maternal neglect, no correlation was found between paternal neglect and maladaptive CERS (β=-0.045, *p* = .470). An examination of the indirect relations via trait EI was examined, it was seen that the relationship between paternal neglect and adaptive (β=-0.011, *p* = .792) became insignificant. While the effect of trait EI is significant, the fact that paternal neglect becomes insignificant shows that trait EI has a full mediator effect on the relationship between paternal neglect and adaptive CERS (F_2,262_= 28.154, *p* < .01, *R* = .421, R^2^ = 0.177), but does not mediate the relationship between paternal neglect maladaptive CERS (F_2,262_= 3.540, *p* < .05, *R* = .162, R^2^ = 0.026). The fit indices for the final mediation model of paternal neglect (א^2^/df = 26.896, *p* < .01; GFI = 1, AGFI = 0.999, NFI = 1, RFI = 999, CFI = 1, RMSEA = 0.000) showed a good fit of the data to the model [67, 68]. The path diagram created for the full paternal model is presented in Fig. 2.

## Discussion

The aim of the present study was to examine the mediating role of trait EI in the relationship between parental neglect and CERS. According to this purpose, the first hypothesis was to examine whether there were associations between parental neglect and trait EI. As a result, both maternal and paternal neglect were found to be negatively associated with trait EI. This finding is in line with a number of findings in the literature, indicating that parental unavailability, including emotional rejection, lack of warmth and emotional support, predicted lower trait EI in children [69]. In addition, parental warmth and nurturance is shown to be associated with children’s emotional knowledge [70], awareness [71] and understanding [72] and empathy [73]. It can be suggested that without parents’ availability and responsiveness, children are deprived of the most important resource for emotional development. Therefore, it is suggested that parental availability contributes to increased insight in children with regard to their ability to observe and understand others’ emotions as well as their own.

In the study, in addition to trait EI, both maternal and paternal neglect were found to be negatively correlated with adaptive CERS, while only maternal neglect was positively correlated with maladaptive CERS. In the literature, the relationship between CERS and parental attitudes has been examined in a number of studies. In a clinical sample of neglected children, Shipman et al. [74] found that compared to their non-maltreated peers, neglected children demonstrated a lower understanding of emotions and fewer adaptive CERS. Studies showing that rejecting and emotionally unavailable parents inhibit their children’s ability to use emotion regulation strategies and cause children to display maladaptive strategies are in line with this finding [75–77]. Emotionally available mothers’ use of refocusing and reappraisal strategies for negative events reduces emotional difficulties in children [78]. In contrast, neglectful mothers often fail to provide positive affect and sensitivity as well as to model and supervise their children for coping with adaptive vs. maladaptive feelings, and their children may have problems coping with emotions such as anger and intolerance of frustration [79]. Accordingly, it has been found that children who have difficulties in emotion regulation develop maladaptive strategies such as catastrophizing and blaming others and themselves to cope with these emotions [80]. Neglected children may consider themselves as responsible for neglect and experience intense feelings of shame [81]. In a study conducted by Gold et al. [82], it was also suggested that maltreated children experience intense feelings of shame, as one of the types of maladaptive CERS, and this situation causes children to blame others. In a study conducted by Çalışkan [83], a relationship was found between maternal rejection behavior and emotion regulation difficulties, and this relationship was found to be particularly evident in rumination strategies. Similarly, other studies have found that individuals who do not perceive their parents as emotionally warm use adaptive CERS less [84, 85]. In the present study, unlike mothers, it was found that paternal neglect did not predict maladaptive CERS in children. It is thought that this difference between mothers and fathers may be related to the fact that fathers’ support and participation in caregiving is less than mothers in Turkish culture. It can be thought that the awareness of fathers about their roles and their tendency to show emotional closeness towards their children affect their children’s ability to understand and manage emotions, whereas in families where fathers are less involved in caregiving, mothers have more positive and negative effects than fathers. This argument also coincides with the study conducted by Uçar [86] on a Turkish sample, which revealed that fathers had less influence on children’s CERS compared to mothers.

The main hypothesis of the study was that the relationship between both maternal and paternal neglect and CERS was mediated by trait EI. Except for the relationship between paternal neglect and maladaptive CERS, this hypothesis was confirmed and trait EI was found to have a full mediator role in both maternal and paternal models, According to this result, it can be argued that higher trait EI may be related to the use of adaptive coping strategies. Similar to the present study, Mikolajczak et al. [87] also revealed that higher trait EI was associated with adaptive CERS and that this association was also valid in the case of stress, fear, sadness, anger, and shame. In another study, Petrides et al. [7] indicated that when confronted with a negative situation, individuals with higher trait EI are inclined to invoke pleasant memories or thoughts in order to face their emotional state, and take actions to handle the problem accordingly, rather than catastrophizing the experience and blaming themselves.

The suggestion that trait EI may reduce the negative impact of neglect needs more focus. According to Goleman [2], individuals with a history of maltreatment in childhood develop the ability to recognize the emotions of others in order to protect themselves from emotional trauma as these individuals become extremely sensitive to emotional stimuli in social situations. While maladaptive patterns occur in many neglected children, the reason why some children develop higher emotionally adaptive skills may be the subject of an interesting but different discussion. However, it can be argued that children with higher trait EI have the ability to understand other people’s emotions by relating the information to their own experiences. Therefore, the emotional abilities they develop while trying to cope with neglect experiences may support the development of adaptive skills such as more positive thinking and the ability to emotionally handle negative situations. Focusing on this suggestion in reverse, whether adaptive CERS predicts trait EI is also an interesting question for future researches according to the results of this study. On the other hand, the positive correlation between adaptive and maladaptive CERS was an unexpected result in the study as it seems reasonable to expect maladaptive CERS to be less, while adaptive CERS is higher in high-trait EI children. This result may suggest that neglected children use all emotion regulation skills, including both adaptive and maladaptive, to cope with their negative emotional experience, but may use adaptive CERS more if their trait EI is higher. It is thought that this finding may extend the results of studies revealing that maltreated children use maladaptive strategies to cope with their negative emotions to a more holistic interpretation [88, 89].

Overall, the findings of this study contribute to the literature by identifying a variable that mediates adaptive cognitive emotion regulation with both maternal and paternal neglect. The fact that maladaptive emotion regulation is associated with maternal neglect, but the same pattern is not evident in fathers, is a context that deserves further study. Despite the strengths, several limitations of the present study should be noted. In the study, while the effect of neglect as a predictive variable was examined, moderator effects other than demographic variables that may have effects on children’s trait EI and CERS were not. Therefore the findings of the mediation analyses must be considered cautiously. Another limitation is that the frequency and time of neglect experiences were not evaluated and the mediating process was based on cross-sectional measures. Therefore, it is not possible to argue that the results related to trait EI and CERS are a direct result of parental neglect. From an ecological perspective, many factors that may be protective may moderate the effect of neglect. In this context, further research may be warranted on the moderated mediation model, which considers protective factors inside and outside the home (i.e. whether there is a person who can replace the parents). In addition, when interpreting the results, it should be taken into account that the data are based on children’s self-reports and the responses about neglect are entirely based on their own subjective perceptions. Future studies should thus consider reports from teachers, social workers or other adult caregivers as well as children. Testing the hypotheses of the present study in a clinical sample can also provide deeper comprehension on the subject.

## Conclusion

The results of the study are particularly significant in two respects. First, while the effects of child abuse is a subject that is frequently studied, the findings of the present study extend the available knowledge on neglect, which is a more vague concept compared to abuse, and therefore much less studied. The other significance is the contribution of the study to the understanding of the concept of trait EI. To the best of the researchers’ knowledge, the present study is the first to reveal the relationship of trait EI and CERS with parental neglect. It is thought that these results may contribute to the comprehension of the dynamics underlying the emotional and cognitive development of children at risk of neglect and provide implications for psychological support. It can be argued that children’s disposition to understand and manage emotions may prevent the destructive effects of neglect and the emergence of maladaptive patterns, as studies indicate that trait EI and adaptive coping strategies may have an important function in protecting children against psychopathological risks [90, 91]. Therefore, it seems important for caregivers and clinicians working with children to support the emotional and cognitive skills of neglected children. In addition to these, it is thought that the findings of the study have implications particularly for educational settings. Children’s success in school depends on emotional intelligence as much as academic intelligence. In particular, trait EI is significant in terms of children’s behavior in emotion-relevant situations when they face stress or a problem among peers. Traumatic experiences such as neglect may negatively affect children’s emotional and behavioral reactions and cognitive regulation skills in the school environment and hinder both their social adaptation and academic success. In this context, school teachers or school mental health professionals should evaluate the processes related to trait EI and cognitive emotion regulation as an indicator in terms of neglect and pay attention to supporting these skills of children at risk of neglect.

## Data Availability

The data can be requested from Utku Beyazit (proz2proz@yaho.com) as an electronic file.
